# Stable Isotope-Resolved Metabolomic Differences between Hormone-Responsive and Triple-Negative Breast Cancer Cell Lines

**DOI:** 10.1155/2018/2063540

**Published:** 2018-09-30

**Authors:** Jason H. Winnike, Delisha A. Stewart, Wimal W. Pathmasiri, Susan L. McRitchie, Susan J. Sumner

**Affiliations:** ^1^Analytical Sciences, David H. Murdock Research Institute, Kannapolis, NC 28081, USA; ^2^NIH Eastern Regional Comprehensive Metabolomics Resource Core, Department of Nutrition, University of North Carolina at Chapel Hill Nutrition Research Institute, Kannapolis, NC 28081, USA

## Abstract

**Purpose:**

To conduct an exploratory study to identify mechanisms that differentiate Luminal A (BT474 and MCF-7) and triple-negative (MDA-MB-231 and MDA-MB-468) breast cancer (BCa) cell lines to potentially provide novel therapeutic targets based on differences in energy utilization.

**Methods:**

Cells were cultured in media containing either [U-^13^C]-glucose or [U-^13^C]-glutamine for 48 hours. Conditioned media and cellular extracts were analyzed by ^1^H and ^13^C NMR spectroscopy.

**Results:**

MCF-7 cells consumed the most glucose, producing the most lactate, demonstrating the greatest Warburg effect-associated energy utilization. BT474 cells had the highest tricarboxylic acid cycle (TCA) activity. The majority of energy utilization patterns in MCF-7 cells were more similar to MDA-MB-468 cells, while the patterns for BT474 cells were more similar to MDA-MB-231 cells. Compared to the Luminal A cell lines, TNBC cell lines consumed more glutamine and less glucose. BT474 and MDA-MB-468 cells produced high amounts of ^13^C-glycine from media [U-^13^C]-glucose which was integrated into glutathione, indicating* de novo* synthesis.

**Conclusions:**

Stable isotopic resolved metabolomics using ^13^C substrates provided mechanistic information about energy utilization that was difficult to interpret using ^1^H data alone. Overall, cell lines that have different hormone receptor status have different energy utilization requirements, even if they are classified by the same clinical BCa subtype; and these differences offer clues about optimizing treatment strategies.

## 1. Introduction

Triple-negative breast cancer (TNBC) is considered to be one of the most aggressive subtypes of BCa based on its clinical characteristics, including increased proliferative rate, high metastatic potential, and shorter survival outcomes (overall and relapse-free) [1–5]. It accounts for 15-20% of all diagnosed BCa cases [6–8] and has an increased prevalence in African American and Asian women (~30%–65%) compared to other ethnicities [9–16]. TNBC is defined by the lack in expression of the common BCa markers, estrogen receptor (ER), progesterone receptor (PR), and epidermal growth factor receptor 2/HER2/neu (HER2) [17], which classify other types of BCa and serve as targets for specific treatments; thus, development of targeted therapies for TNBC has been severely hampered. Innovative approaches that identify relevant targets could lead to new therapies that could augment the efficacy of current standard-of-care surgical, chemotherapeutic, or radiation-dependent interventions [18–20].

Now an established hallmark of cancer, aberrant energy metabolism has provided a wealth of information on how tumor cells initiate, progress, and spread [21, 22]. For example, measurement of glycolytic activity is used to identify key metabolic perturbations that significantly impact tumor cell growth and viability, by comparing alterations in glucose uptake and lactate production, as well as other rate-limiting enzymes involved in the endogenous pathways. Another important essential amino acid that has shifted some attention from glucose as a primary energy source in the past few years is glutamine [23] and the process of glutaminolysis [24], which can provide significant energy needs (i.e., NADPH) of rapidly proliferating cells [25]. Metabolomics methods employing stable isotope labeled compounds, such as ^13^C-glucose or ^13^C-glutamine, can be used to interrogate the energetic flux that cancer cells depend on to thrive and avoid host programs for destruction.

In a previous study [26], we compared the metabolic and inflammatory responses of these hormone receptor-positive (Luminal A) and TNBC cell lines following treatment with the widely used chemotherapeutic paclitaxel (Taxol®). There, we showed differences between both the cells classified by the same subtype (BT474 versus MCF-7 and MDA-MB-231 versus MDA-MB-468) and between the two subtype cell models (Luminal A versus TNBC) [26]. Based on our findings, we felt further investigation of the metabolic differences was warranted. This current study presents results to help better understand metabolic differences of these two cell models of different hormone receptor status BCa subtypes. This may identify new TNBC targets, reveal potential points for modulation, and ultimately improve the ability to design therapeutics that disrupt their primary energy utilization paradigms [27].

## 2. Materials and Methods

### 2.1. Cell Culture and Treatment

Luminal A BCa cell lines (BT474 and MCF-7) and TNBC cell lines (MDA-MB-231 and MDA-MB-468) were purchased from ATCC and cultured in DMEM media (Gibco/Life Technologies) supplemented with 10% FBS and 1% antibiotic/antimycotic. All ATCC cell lines undergo authentication tests (i.e., for mycoplasma negativity) during the accessioning process, which is described in the online ATCC brochure Maintaining High Standards in Cell Culture (http://www.atcc.org/). Cells were maintained in culture, incubated under humidified conditions at 37°C in 5% CO_2_.

[U-^13^C]-Glucose was purchased from Cambridge Isotope Laboratories (Tewksbury, MA, USA) and [U-^13^C]-glutamine was purchased from Sigma-Aldrich (St. Louis, MO, USA). Each cell line was plated on 10 cm dishes with DMEM growth media containing 4.5 g/L glucose + 2 mM glutamine (Gibco) for 24 hr. Media was removed and cells were washed three times with sterile 1X PBS and treated for 48 hr in regular high glucose DMEM, DMEM (-glucose) supplemented with [U-^13^C]-glucose or DMEM (-glutamine) supplemented with [U-^13^C]-glutamine. Following incubation, 1 mL media aliquots were collected and stored at −80°C. Cells remaining on dishes were washed three times with ice-cold 1X PBS and extracted using a modified Folch method [28, 29]. Briefly, cells were quenched with 50:50 ice-cold acetonitrile:water, scraped off dishes, and collected into 15 mL tubes containing zirconia beads. Cold chloroform was added and each tube was vigorously vortexed on a multitube vortexer for three 30 sec pulses. Tubes were centrifuged at 3,700 rpm for 60 min at 4°C, and the aqueous fractions were transferred to cryotubes, while the organic fractions were collected into glass vials. The remaining protein layer and residual aqueous and lipid layers were transferred to Lo-Bind microcentrifuge tubes, cold chloroform:methanol (2:1) was added, and the tubes were quickly vortexed and then centrifuged at 15,000 rpm for 20 min at 4°C. The remaining aqueous and lipid fractions were transferred into collection tubes indicated above, and the protein pellets were dried for 20 min on a Speedvac (no heat) and weighed. All samples were stored at −80°C except for the aqueous fractions which were immediately lyophilized to dryness and then stored at −80°C until preparation for NMR analysis. Each condition was performed in triplicate.

### 2.2. NMR Sample Preparation and Data Acquisition

Media samples were removed from −80°C and thawed at 4°C. Samples were vortexed briefly and centrifuged at 18,000 rcf and 4°C for 15 min and a 200 *μ*L aliquot was removed to a microcentrifuge tube. Next, 1 mL of ice-cold methanol:chloroform extraction solvent (3:1) was added and samples were vortexed for 30 sec and centrifuged at the same conditions listed above. Supernatants were transferred to new microcentrifuge tubes and dried overnight under a gentle flow of N_2_. Samples were reconstituted in 700 *μ*L of a deuterium oxide (D_2_O, Aldrich, St. Louis, MO, USA) solution containing 0.6 mM 4,4-dimethyl-4-silapentane-1-sulfonic acid (DSS-D6, Chemical Shift Indicator), 0.6 mM Imidazole (pH indicator), and 0.2% NaN_3_ and vortexed and centrifuged at the same conditions listed above. Following centrifugation, 600 *μ*L aliquots were transferred to 5 mm NMR tubes for data acquisition. NMR spectra were acquired on a Bruker Avance III 600 MHz NMR spectrometer (Bruker-Biospin, Rheinstetten, Germany) using a cryogenically cooled 5 mm ATMA probe at 25°C. For ^1^H spectra, a 1D NOESY pulse sequence (noesypr1d) with water presaturation during the 2 sec relaxation delay and 100 ms mixing time was used, and 128 transients were collected into 16k data points with a spectral width of 6602.1 Hz (11 ppm) and an acquisition time of 2.48 sec.

Lyophilized cellular extracts were reconstituted in the same NMR solution as used for the conditioned media. The samples were vortexed and centrifuged at 12,000 rcf for 3 min; then a 600 *μ*L aliquot of each sample supernatant was transferred into 5 mm NMR tubes (Bruker-BioSpin, Switzerland) for data acquisition. For ^1^H spectra, the same 1D NOESY pulse sequence as described in the previous paragraph was used, except the number of transients was increased from 128 to 256. For ^13^C spectra, a 1D ^13^C pulse sequence (zgpg30) was used with a 2 s relaxation delay and 512 transients were collected into 32k points, with a spectral width of 36057.7 Hz (239 ppm) and an acquisition time of 0.9088 sec. Additionally, two-dimensional NMR spectra were acquired on a few select samples to aid in peak identification. These 2D-HSQC spectra were acquired at 25°C using 16 transients, 256 points in the ^13^C dimension, and 512 points in the ^1^H dimension. The relaxation delay was 1.5 s, spectral widths were 8012.8 Hz (13.4 ppm) and 24997.3 Hz (165.6 ppm) for ^1^H and ^13^C, respectively, and the acquisition time was 0.0639 s.

### 2.3. NMR Data Analysis

All spectra were imported into MestReNova 10.0.1 (Mestrelab Sesearch SL, Santiago de Compostela, Spain) for processing. ^1^H spectra were zero-filled to 64k points, apodized (0.5 Hz for media and 1 Hz for extract), and Fourier transformed. Spectra were manually phased and baseline corrected with a linear first-degree polynomial and referenced to DSS. The peaks in the spectra were then globally deconvoluted using the Global Spectral Deconvolution (GSD) peak picking routine (2 fitting cycles, optimized for average peaks). Peak lists containing chemical shifts and peak area were exported to Microsoft Excel (Microsoft Corporation, Redmond, WA) for further analysis. Peak areas corresponding to metabolites were identified, and using the peak area of the known concentration of DSS, the relative concentration of metabolites was calculated. Protein pellet masses were used for normalization of metabolite concentration data. Carbon spectra were zero-filled to 64k points, apodized with a 10 Hz exponential curve, and Fourier transformed. Spectra were manually phased and baseline corrected with a 13-degree polynomial and referenced to DSS. The peaks in the spectra were then manually integrated and peak lists were exported to Microsoft Excel for further analysis. Peak areas corresponding to identified metabolites were measured for determining relative concentrations and protein pellet masses were used to normalize the metabolite semiquantifications. Media production and consumption values were calculated using the ^12^C media experimental replicates, with the difference from fresh unused media being normalized to pellet weight. As mentioned above, each condition was measured in triplicate and therefore all measurements of intracellular concentrations and media production and consumption are presented as averages with standard deviations indicated by parentheses in the first two tables. Fold changes were calculated in the third table for all pairwise comparisons between cell lines, relative to each cell line. Hypothesis testing was not conducted due to the small sample size in this pilot study. All raw metabolomics data is publicly available at the NIH Common Fund Metabolomics Data Repository and Coordinating Center website, Metabolomics Workbench: http://www.metabolomicsworkbench.org/data.

## 3. Results and Discussion

### 3.1. Glycolysis, Glutaminolysis, and the Citric Acid Cycle

**Figure 1:**
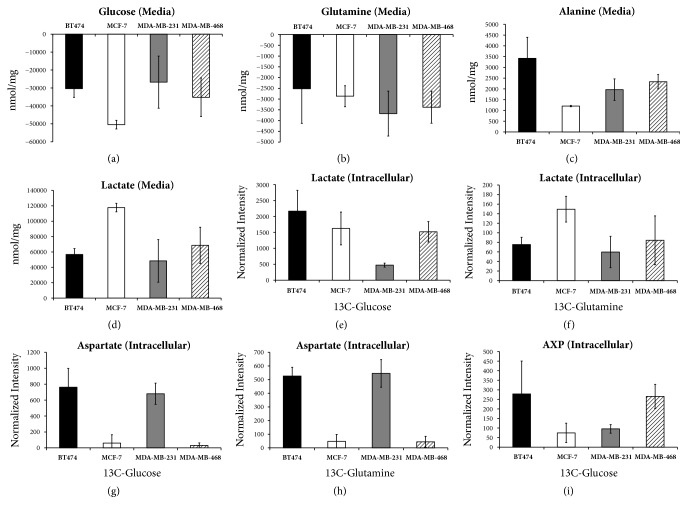
Intracellular and media levels for selected metabolites of interest. Glucose and glutamine values indicate media consumption. AXP is the sum of AMP, ADP, and ATP which have overlapping peaks that are difficult to differentiate in this case. Graphs are presented as averages of three replicate measurements for each condition/cell line with the error bars reflecting standard deviation between replicates.

Table 1 shows some of the differences of glucose and glutamine utilization by each cell line, based on integration of both ^1^H and ^13^C carbon NMR signals from the cellular extracts and cultured media. Across the four cell lines, glycolytic-dependent differences were immediately evident upon review of the spectral data (Supplemental Figure 1A) and likewise for glutamine peaks in the ^13^C-glutamine spectra (Supplemental Figure 1B). Luminal A MCF-7 cells used the most glucose and, not surprisingly, produced the most lactate, indicating the Warburg effect [30] being a very active energy utilization mechanism (Figures 1(a)–1(d)). Additionally, very little ^13^C-aspartate is produced from [U-^13^C]-glucose or [U-^13^C]-glutamine, suggesting little TCA activity in the cultured cells. Similarly, this small production of aspartate was also seen in triple-negative MDA-MB-468 cells (Figures 1(g) and 1(h)).

On the other hand, the BT474 Luminal A cell line had high alanine and low lactate production, seen in both the intracellular and media samples (Figures 1(c) and 1(d)), in addition to high ^13^C-aspartate production from [U-^13^C]-glucose or [U-^13^C]-glutamine, indicative of high TCA activity. Their lower glucose and glutamine utilization may be indicative of this more efficient use of these energy substrates. A similarly high level of ^13^C-aspartate production from [U-^13^C]-glucose and [U-^13^C]-glutamine was also seen in the MDA-MB-231 (TNBC) cell line (Figures 1(g) and 1(h)), and as expected we saw a smaller production of lactate in the media, compared to the other TNBC cells (Figures 1(d) and 1(e)). There was also a higher catabolism of the media glutamine in the MDA-MB-231 cells (Table 2). Thus, the high ^13^C-aspartate, low media lactate, lower glucose catabolism, and higher glutamine catabolism seem to indicate increased TCA activity and lower anaerobic glycolysis in these cell lines that represent different subtypes of the disease, and our data provide a different way to classify them. Interestingly, in our previously published study, we described the responses of these four cell lines to treatment with the chemotherapeutic drug paclitaxel, showing by unsupervised multivariate analysis [26] that, in the absence of drug, MCF-7 and MDA-MB-468 cells cluster together and likewise BT474 and MDA-MB-231. This observation seems puzzling because it is assumed that the cell lines are most similar based on hormone receptor status and therefore by clinical/molecular subtype classification. In fact, in two previous studies, comparing BCa cell lines across different subtypes, we have shown the lines cluster together by subtype based on both genomic profiles [31] and expression pattern changes after stromal macrophage interaction [5]. Thus, because both BT474 (HER2^+^) and MCF-7 (HER2^−^) cells express ER and PR and are accepted as representative of the Luminal A subtype, it was not expected that BT474 would be more metabolically similar to the MDA-MB-231, triple-negative line and likewise the MCF-7 luminal cells more metabolically similar to the triple-negative MDA-MB-468 cells. Our data suggests that, in addition to a potentially new way to classify BCa cell lines, HER2 status may contribute a more significant metabolic role toward energy-dependent mechanisms. These findings may be particularly relevant when considering response to different types of treatments, especially when developing novel targeted strategies.

### 3.2. Nucleotide Metabolism

**Figure 2:**
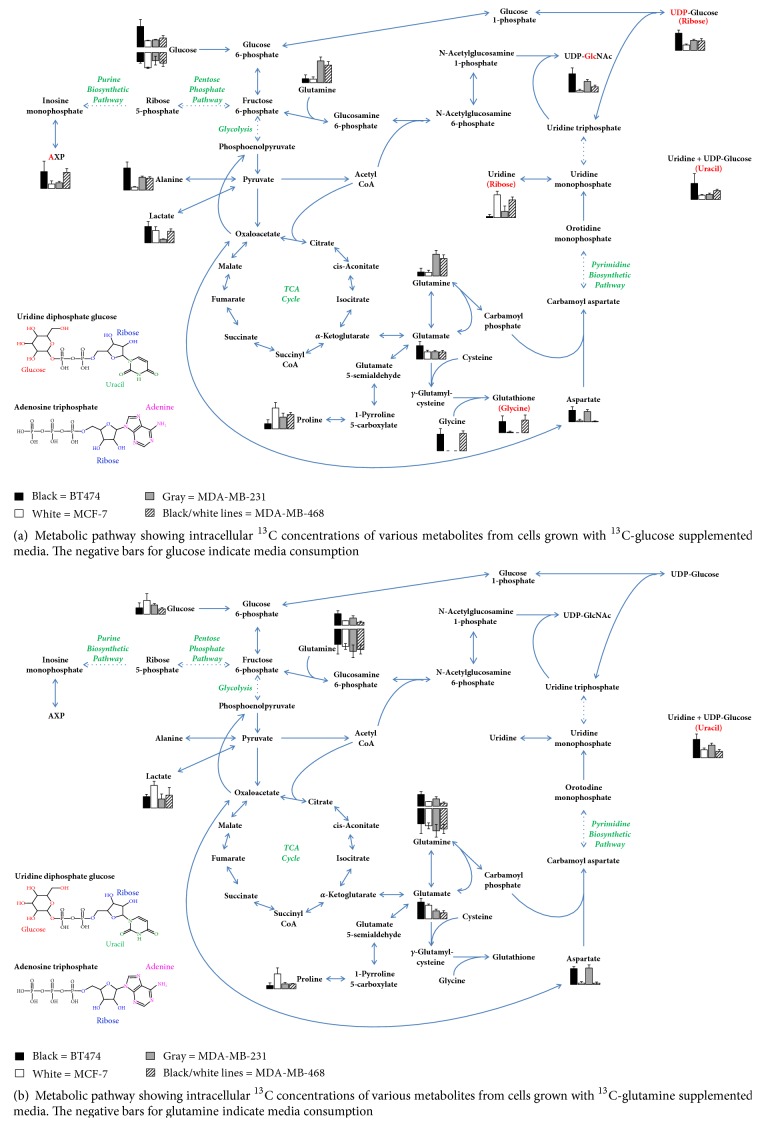


The following nucleotides could be seen by either ^13^C or ^1^H NMR spectroscopy: adenosine mono-, di-, or triphosphate (AXP) (Figure 1(i)), uridine, UDP-glucose, and UDP-N-acetylglucosamine (UDP-GlcNAc). Both UDP-glucose and UDP-N-acetylglucosamine production (measured via ^13^C enrichment in ribose and glucosamine moieties, respectively) are mirrored by opposite ^13^C enrichment trends in uridine (measured via ^13^C enrichment in the ribose moiety). Thus, it appears that the uridine pool very closely reflects the production of nucleotide sugars in the sense that, as its pool decreases, it is utilized for nucleotide sugar production.  Table 3 indicates the fold-change differences between intracellular, produced, and consumed metabolite concentrations from Tables 1 and 2, while Figure 2 shows an overall picture of these key pathways and relative metabolite changes.

The low concentration of ^13^C labeled uridine and high concentrations of labeled AXP as well as the nucleotide sugars from the [U-^13^C]-glucose in the media of BT474 cells may be a consequence of the seemingly higher aerobic TCA activity seen in these cells compared to the other three lines. Additionally, a much higher intracellular concentration of creatine was seen in BT474, which catalyzes the formation of ATP from ADP. Since anaerobic glycolysis is the less efficient way of generating energy from glucose, the other cell lines may be prioritizing glucose for anaerobic catabolism. This appears to be the case for the MCF-7 cells. Regarding uridine, the nucleotide sugars, and AXP, MCF-7 cells had a completely opposite trend compared to the BT474 cells. Additionally, glucose consumption, total lactate production, and ^13^C aspartate concentrations (from both [U-^13^C]-glucose and [U-^13^C]-glutamine) have opposite trends for these two Luminal A lines, demonstrating the MCF-7 cells exhibit higher anaerobic glycolysis and lower TCA activity than BT474 cells.

It is interesting to note that the two TNBC cell lines, like in other aspects of metabolism, also appear to be metabolically distinct with regard to nucleotide and nucleotide sugar metabolism. MDA-MB-231 cells had low ^13^C enrichment from media [U-^13^C]-glucose in uridine and AXP while it was higher for the nucleotide sugars, while the opposite was mostly true for MDA-MB-468 cells. In our related study on treatment response differences, we used untargeted metabolomics and evaluated circulating inflammatory biomarker data to identify differences between these lines, based on the ethnic origin of the women the lines were established from. MDA-MB-468 cells were established from a sample taken from an African American woman, while MDA-MB-231 cells were established from a Caucasian woman [32–36]. Because TNBC has demonstrated health disparities, by ethnicity, in terms of incidence, mortality, and outcomes linked to responsiveness to treatment [2, 6, 9–15, 37–41], it is reasonable that we continue to see differences between the lines at the level of energy utilization.

### 3.3. Amino Acid Metabolism

There was similarly high ^13^C-glycine production from media [U-^13^C]-glucose in both BT474 and MDA-MB-468 cells and little to no detectible production in MCF-7 and MDA-MB-231 cells. Predictably, this trend was also seen in the ^13^C enrichment of the glycine moiety of glutathione (*γ*-glu-cys-gly). Unfortunately, due to peak overlap, we were not able to discern the glutamate moiety of glutathione from glutamine or free glutamate in the ^13^C spectra. This* de novo* glutathione production in the BT474 and MDA-MB-468 cells may be indicative of their capacity to respond to oxidative stress and overcome hypoxic microenvironment conditions, processes which have been linked to decreased treatment responsivity, increased metastatic potential, and antiapoptotic function [42–44].

There were other differences in amino acid concentrations and production beyond what has been discussed. Of particular interest is the observation that the consumption of the branched chain amino acids (isoleucine, leucine, and valine) from the media occurs in a trend roughly opposite to that of glucose. For instance, MCF-7 cells have the highest glucose consumption but the lowest valine consumption, whereas BT474 and MDA-MB-231 cells have the lowest glucose consumption but the highest valine consumption. Consumption of other aromatic amino acids or methionine was unremarkable. Intracellular concentrations of both aromatic and branched chain amino acids follow roughly the same trend, where MDA-MB-231 and BT474 had the highest concentration, and the TNBC line had the highest concentration, but MCF-7 and MDA-MB-468 cells had lower concentrations by comparison. Overall, intracellular concentrations mirrored uptake from the media.

## 4. Conclusions

The addition of ^13^C substrates to the media uncovered many findings that were not possible using “traditional” methodologies involving ^12^C substrates. Since the majority of lactate production is from glucose and a much smaller amount is from glutamine, use of ^13^C-glutamine in the media allows the production of lactate from glutamine to be observed. Similarly, production of both glutamate and glutamine from glucose, as well as production of glucose from glutamine, can be observed using media containing ^13^C-glucose or ^13^C-glutamine, respectively. The ^13^C measurements, which represent production from the ^13^C substrate, differ from their ^12^C counterparts, which represent total pools of the given metabolite. Additionally,* de novo* synthesis of many compounds that are recycled or partially degraded can be observed using ^13^C substrates. ^13^C measurements of AXP and GSH from media containing ^13^C-glucose are different from their ^12^C counterparts. Thus, the ^13^C measurements would be indicative of* de novo* synthesis rather than the total pools of these heavily recycled metabolites. Finally, using the ^13^C experimental procedure, we were able to measure metabolites, notably proline and uridine (Figure 2), that would have been otherwise difficult for concentration fit, because they have ^1^H NMR spectral peaks that are masked by other larger peaks.

Our data demonstrate the utility of stable isotope-resolved metabolomics to differentiate BCa cells lines, classified by the same clinical subtype based on hormone receptor status, by differences in energy utilization. These characteristic differences should be considered when designing effective targeted treatment strategies. To that end, future studies with sufficient power will statistically test the differences observed in metabolites that play a role in cellular energetics. Next, we could determine how differences in energy utilization in BCa cell line models correlate with basal energy utilization requirements using normal mammary cells, to identify metabolically driven mechanisms and potentially novel therapeutic targets.

## Figures and Tables

**Table 1:** Semiquantitative concentrations of intracellular metabolites*∗*.

	**BT474**		**MCF-7**		**MDA-MD-231**		**MDA-MB-468**	
**Metabolites**	^*13*^ *C Glc*	^*13*^ *C Gln*	^*13*^ *C Glc*	^*13*^ *C Gln*	^*13*^ *C Glc*	^*13*^ *C Gln*	^*13*^ *C Glc*	^*13*^ *C Gln*
**Adenosine**	229.2 (210.1)	50.1 (60.4)	28.1 (35)	0 (0)	28.6 (32)	2.8 (4.8)	296.2 (39.7)	44.9 (17.2)
**Alanine**	7293.5 (2003)	56.5 (97.9)	981.1 (191.1)	13.3 (23.1)	4225.2 (389.6)	155 (158.4)	3947.3 (468.7)	25.9 (26.8)
**Aspartate**	762.1 (236.5)	526.4 (63.8)	60.4 (104.6)	47.7 (50.9)	679.5 (133)	545.7 (101.3)	30.8 (31.5)	44.7 (39.9)
**AXP**	278.4 (171.9)	0 (0)	74.5 (50.7)	23.3 (10.6)	95.6 (22.4)	35.5 (27.9)	265.4 (62.9)	54.2 (36.8)
**Glucose**	6791.1 (2295.2)	422.3 (327)	2103.3 (128.9)	914.6 (440.3)	2346 (168.3)	607.9 (96.1)	2989.7 (743.3)	353 (83.1)
**Glutamate**	1765.2 (531.5)	1103.6 (242.6)	956 (227.8)	894.6 (88.5)	972.3 (163.2)	528.8 (120.7)	945.3 (205.8)	398.9 (145.5)
**Glutamine**	534.9 (497.9)	7697.9 (1913.5)	483.4 (285.1)	3030 (128.1)	2879.8 (512)	4812.5 (1445.6)	2318 (474.2)	2081.8 (667.6)
**Glycine**	1098.1 (374.1)	72 (124.7)	0 (0)	7.8 (13.6)	0 (0)	0 (0)	1145.3 (169.4)	14.5 (25.2)
**Lactate**	2167.3 (653.3)	75.4 (15.1)	1623.7 (515.1)	149.3 (26.9)	472.8 (63.5)	59.8 (32.7)	1518.7 (320.8)	84.3 (51)
**Glutathione**	148.3 (72.4)	23.8 (41.3)	7.8 (13.5)	1.9 (3.4)	0 (0)	0 (0)	165.9 (67.7)	6.3 (11)
**Proline **	170.2 (131.3)	108.5 (86.9)	689.4 (183.5)	491 (198)	390.5 (128.8)	160.9 (45.4)	472.5 (68.1)	161.6 (14.7)
**UDP-GlcNAc**	1212.9 (387.9)	146 (127.7)	100.1 (86.8)	63.5 (12.5)	705.9 (126.7)	24.2 (4.6)	357.4 (107.1)	26.4 (4)
**UDP-Glucose**	1422.4 (267.1)	21.9 (38)	357.2 (79.9)	21 (18.4)	719.4 (80.6)	43.2 (68.4)	690.6 (93)	82.1 (16.5)
**Uridine**	15.9 (27.5)	18.4 (31.9)	302.8 (46)	5.8 (10)	82.9 (71.4)	33.1 (27.8)	234.5 (37.9)	32 (46.2)
**Uridine/UDP-G**	2275.9 (409.5)	86.5 (149.8)	721.7 (122.3)	0 (0)	1331.4 (127.8)	9.7 (16.9)	1247.3 (310.5)	32.8 (38.6)

*∗* Semiquantitative values have been normalized to pellet weight and are unitless. Values represent average among three replicates and the standard deviation is in parentheses.

**Table 2:** Metabolite consumption and production in conditioned medias.

**Metabolite**	**BT474**	**MCF-7**	**MDA-MB-231**	**MDA-MB-468**
**Glucose**	30381.9 (4849.4)	50449.5 (2418.9)	26775.3 (14543.3)	35193.2 (10767.5)
**Glutamine**	2520.4 (1605.3)	2865.5 (487.3)	3673.5 (1042.1)	3376.1 (746)
**Isoleucine**	1088.1 (194.2)	504.3 (156.5)	1091.9 (94.3)	843.4 (142.3)
**Leucine**	1897.8 (392.2)	1341.3 (197.9)	1538.6 (244.2)	1488.2 (126.3)
**Methionine**	330.5 (102.2)	174.2 (160.1)	253.9 (81.2)	252.2 (38.8)
**Phenylalanine**	55.0 (52.9)	47.5 (47.2)	156.5 (159.2)	184.5 (80.1)
**Threonine**	133.1 (133.3)	187.5 (163.7)	368.6 (324.3)	447.5 (140.6)
**Tryptophan**	31.9 (35.3)	33.1 (29.3)	50.1 (22.9)	41.4 (16.6)
**Tyrosine**	158.6 (95.6)	186.8 (52.2)	208.2 (122.5)	215.4 (87.4)
**Valine**	1141.9 (250.6)	671.5 (162.1)	1056.7 (124.9)	858.3 (16)

*2-Hydroxy-butyrate*	388.4 (173.5)	223.3 (51.2)	513.1 (150.9)	368.8 (172.5)
*Acetate*	144.4 (67.4)	95.2 (30)	203.2 (124.5)	115.6 (124.6)
*Alanine*	3420.3 (978.3)	1200.9 (29.8)	1966.6 (498.4)	2333.5 (332.8)
*Glutamate*	1010.2 (390.5)	1385.2 (306.5)	287.1 (124.8)	215.9 (302.8)
*Lactate*	56700 (7688)	117705.8 (5518)	48395.6 (27755.1)	68584.2 (23542.9)

Averages (in nmol/mg) calculated from ^12^C signals. Standard deviations in parentheses normalized to dried pellet weight. Consumption is reflected by **bolded** metabolites. Production is reflected by *italicized* metabolites.

**Table 3:** Fold-change differences for cell line intracellular (^13^C), consumption, and production (^12^C) concentration comparisons.

**NMR signal**	^**13**^ **C Glc**				^**13**^ **C Gln**				^**12**^ **C**			
**Cell lines**	**BT474**	**MCF-7**	**MDA-MD-231**	**MDA-MB-468**	**BT474**	**MCF-7**	**MDA-MD-231**	**MDA-MB-468**	**BT474**	**MCF-7**	**MDA-MB-231**	**MDA-MB-468**

**Metabolites**	**2-Hydroxy-butyrate**											

relative to BT474									*1.00*	-4.25	*1.32*	*-0.50*
relative to MCF-7									*1.74*	*1.00*	2.30	*1.65*
relative to MDA-MB-231									-2.43	-5.65	*1.00*	-2.81
relative to MDA-MB-468									*1.05*	-3.95	*1.39*	*1.00*

	**Acetate**											

relative to BT474									*1.00*	-3.41	*1.41*	*-1.99*
relative to MCF-7									*1.52*	*1.00*	2.13	*1.21*
relative to MDA-MB-231									-2.89	-5.31	*1.00*	-4.31
relative to MDA-MB-468									*1.25*	*-1.76*	*1.76*	*1.00*

	**Adenosine**											

relative to BT474	*1.00*	-8.77	-8.75	*1.29*	*1.00*	-9.80	-9.44	*-1.04*				
relative to MCF-7	8.16	*1.00*	*1.02*	10.54	50.10	*1.00*	2.80	44.90				
relative to MDA-MB-231	8.01	*-0.17*	*1.00*	10.36	17.89	-6.43	*1.00*	16.04				
relative to MDA-MB-468	-2.26	-9.05	-9.03	*1.00*	*1.12*	-9.78	-9.38	*1.00*				

	**Alanine**											

relative to BT474	*1.00*	-8.65	-4.21	-4.59	*1.00*	-7.65	2.74	-5.42	*1.00*	-6.49	-4.25	-3.18
relative to MCF-7	7.43	*1.00*	4.31	4.02	4.25	*1.00*	11.65	*1.95*	2.85	*1.00*	*1.64*	*1.94*
relative to MDA-MB-231	*1.73*	-7.68	*1.00*	*-0.66*	-6.35	-9.14	*1.00*	-8.33	*1.74*	-3.89	*1.00*	*1.19*
relative to MDA-MB-468	*1.85*	-7.51	*1.07*	*1.00*	2.18	-4.86	5.98	*1.00*	*1.47*	-4.85	*-1.57*	*1.00*

	**Aspartate**											

relative to BT474	*1.00*	-9.21	*-1.08*	-9.60	*1.00*	-9.09	*1.04*	-9.15				
relative to MCF-7	12.62	*1.00*	11.25	-4.90	11.04	*1.00*	11.44	*-0.63*				
relative to MDA-MB-231	*1.12*	-9.11	*1.00*	-9.55	*-0.35*	-9.13	*1.00*	-9.18				
relative to MDA-MB-468	24.74	*1.96*	22.06	*1.00*	11.78	*1.07*	12.21	*1.00*				

	**AXP**											

relative to BT474	*1.00*	-7.32	-6.57	*-0.47*	*1.00*	23.30	35.50	54.20				
relative to MCF-7	3.74	*1.00*	*1.28*	3.56	-9.57	*1.00*	*1.52*	2.33				
relative to MDA-MB-231	2.91	-2.21	*1.00*	2.78	-9.72	-3.44	*1.00*	*1.53*				
relative to MDA-MB-468	*1.05*	-7.19	-6.40	*1.00*	-9.82	-5.70	-3.45	*1.00*				

	**Glucose**											

relative to BT474	*1.00*	-6.90	-6.55	-5.60	*1.00*	2.17	*1.44*	*-1.64*	*1.00*	*1.66*	*-1.19*	*1.16*
relative to MCF-7	3.23	*1.00*	*1.12*	*1.42*	-5.38	*1.00*	-3.35	*-0.47*	-3.98	*1.00*	-4.69	-3.02
relative to MDA-MB-231	2.89	*0.90*	*1.00*	*1.27*	-3.05	*1.50*	*1.00*	*-0.47*	*1.13*	*1.88*	*1.00*	*1.31*
relative to MDA-MB-468	2.27	-2.96	-2.15	*1.00*	*1.20*	2.59	*1.72*	*1.00*	*-1.37*	*1.43*	-2.39	*1.00*

	**Glutamate**											

relative to BT474	*1.00*	-4.58	-4.49	-4.64	*1.00*	*-1.89*	-5.21	-6.39	*1.00*	*1.37*	-7.16	-7.86
relative to MCF-7	*1.85*	*1.00*	*1.02*	*-0.11*	*1.23*	*1.00*	-4.09	-5.54	-2.71	*1.00*	-7.93	-8.44
relative to MDA-MB-231	*1.82*	*-0.17*	*1.00*	*-0.28*	2.09	*1.69*	*1.00*	-2.46	3.52	4.82	*1.00*	-2.48
relative to MDA-MB-468	*1.87*	*1.01*	*1.03*	*1.00*	2.77	2.24	*1.33*	*1.00*	4.68	6.42	*1.33*	*1.00*

	**Glutamine**											

relative to BT474	*1.00*	*-0.96*	5.38	4.33	*1.00*	-6.06	-3.75	-7.30	*1.00*	*1.14*	*1.46*	*1.34*
relative to MCF-7	*1.11*	*1.00*	5.96	4.80	2.54	*1.00*	*1.59*	-3.13	*-1.20*	*1.00*	*1.28*	*1.18*
relative to MDA-MB-231	-8.14	-8.32	*1.00*	*-1.95*	*1.60*	-3.70	*1.00*	-5.67	-3.14	-2.20	*1.00*	*-0.81*
relative to MDA-MB-468	-7.69	-7.91	*1.24*	*1.00*	3.70	*1.46*	2.31	*1.00*	-2.53	*-1.51*	*1.09*	*1.00*

	**Glutathione**											

relative to BT474	*1.00*	-9.47	-9.93	*1.12*	*1.00*	-9.20	-9.58	-7.35				
relative to MCF-7	19.01	*1.00*	-8.72	21.27	12.53	*1.00*	-4.74	3.32				
relative to MDA-MB-231	148.30	7.80	*1.00*	165.90	23.80	*1.90*	*1.00*	6.30				
relative to MDA-MB-468	*-1.06*	-9.53	-9.94	*1.00*	3.78	-6.98	-8.41	*1.00*				

	**Glycine**											

relative to BT474	*1.00*	-9.99	-9.99	*1.04*	*1.00*	-8.92	-9.86	-7.99				
relative to MCF-7	1098.10	*1.00*	*1.00*	1145.30	9.23	*1.00*	-8.72	*1.86*				
relative to MDA-MB-231	1098.10	*1.00*	*1.00*	1145.30	72.00	7.80	*1.00*	14.50				
relative to MDA-MB-468	*-0.41*	-9.99	-9.99	*1.00*	4.97	-4.62	-9.31	*1.00*				

	**Isoleucine**											

relative to BT474									*1.00*	-5.37	*1.00*	-2.25
relative to MCF-7									2.16	*1.00*	2.17	*1.67*
relative to MDA-MB-231									*1.00*	-5.38	*1.00*	-2.28
relative to MDA-MB-468									*1.29*	-4.02	*1.29*	*1.00*

	**Lactate**											

relative to BT474	*1.00*	-2.51	-7.82	-2.99	*1.00*	*1.98*	-2.07	*1.12*	*1.00*	2.08	*-1.46*	*1.21*
relative to MCF-7	*1.33*	*1.00*	-7.09	*-0.65*	-4.95	*1.00*	-5.99	-4.35	-5.18	1.00	-5.89	-4.17
relative to MDA-MB-231	4.58	3.43	*1.00*	3.21	*1.26*	2.50	*1.00*	*1.41*	*1.17*	2.43	*1.00*	*1.42*
relative to MDA-MB-468	*1.43*	*1.07*	-6.89	*1.00*	*-1.06*	*1.77*	-2.91	*1.00*	*-1.73*	*1.72*	-2.94	*1.00*

	**Leucine**											

relative to BT474									*1.00*	-2.93	*-1.89*	-2.16
relative to MCF-7									*1.41*	*1.00*	*1.15*	*1.11*
relative to MDA-MB-231									*1.23*	*-1.28*	*1.00*	*-0.33*
relative to MDA-MB-468									*1.28*	*-0.99*	*1.03*	*1.00*

	**Methionine**											

relative to BT474									*1.00*	-4.73	-2.32	-2.37
relative to MCF-7									*1.90*	*1.00*	*1.46*	*1.45*
relative to MDA-MB-231									*1.30*	-3.14	*1.00*	*-0.07*
relative to MDA-MB-468									*1.31*	-3.09	*1.01*	*1.00*

	**Phenylalanine**											

relative to BT474									*1.00*	*-1.36*	2.85	3.35
relative to MCF-7									*1.16*	*1.00*	3.29	3.88
relative to MDA-MB-231									-6.49	-6.96	*1.00*	*1.18*
relative to MDA-MB-468									-7.02	-7.43	*-1.52*	*1.00*

	**Proline**											

relative to BT474	*1.00*	4.05	2.29	2.78	*1.00*	4.53	*1.48*	*1.49*				
relative to MCF-7	-7.53	*1.00*	-4.34	-3.15	-7.79	*1.00*	-6.72	-6.71				
relative to MDA-MB-231	-5.64	*1.77*	*1.00*	*1.21*	-3.26	3.05	*1.00*	*1.00*				
relative to MDA-MB-468	-6.40	*1.46*	*-1.74*	*1.00*	-3.29	3.04	*1.00*	*1.00*				

	**Threonine**											

relative to BT474									*1.00*	*1.41*	2.77	3.36
relative to MCF-7									-2.90	*1.00*	*1.97*	2.39
relative to MDA-MB-231									-6.39	-4.91	*1.00*	*1.21*
relative to MDA-MB-468									-7.03	-5.81	*-1.76*	*1.00*

	**Tryptophan**											

relative to BT474									*1.00*	*1.04*	*1.57*	*1.30*
relative to MCF-7									*-0.36*	*1.00*	*1.51*	*1.25*
relative to MDA-MB-231									-3.63	-3.39	*1.00*	*-1.74*
relative to MDA-MB-468									-2.29	-2.00	*1.21*	*1.00*

	**Tyrosine**											

relative to BT474									*1.00*	*1.18*	*1.31*	*1.36*
relative to MCF-7									*-1.51*	*1.00*	*1.11*	*1.15*
relative to MDA-MB-231									-2.38	*-1.03*	*1.00*	*1.03*
relative to MDA-MB-468									-2.64	*-1.33*	*-0.33*	*1.00*

	**UDP-GlcNAc**											

relative to BT474	*1.00*	-9.17	-4.18	-7.05	*1.00*	-5.65	-8.34	-8.19				
relative to MCF-7	12.12	*1.00*	7.05	3.57	2.30	*1.00*	-6.19	-5.84				
relative to MDA-MB-231	*1.72*	-8.58	*1.00*	-4.94	6.03	2.62	*1.00*	*1.09*				
relative to MDA-MB-468	3.39	-7.20	*1.98*	*1.00*	5.53	2.41	*-0.83*	*1.00*				

	**UDP-Glucose**											

relative to BT474	*1.00*	-7.49	-4.94	-5.14	*1.00*	*-0.41*	*1.97*	3.75				
relative to MCF-7	3.98	*1.00*	2.01	*1.93*	*1.04*	*1.00*	2.06	3.91				
relative to MDA-MB-231	*1.98*	-5.03	*1.00*	*-0.40*	-4.93	-5.14	*1.00*	*1.90*				
relative to MDA-MB-468	2.06	-4.83	*1.04*	*1.00*	-7.33	-7.44	-4.74	*1.00*				

	**Uridine**											

relative to BT474	*1.00*	19.04	5.21	14.75	*1.00*	-6.85	*1.80*	*1.74*				
relative to MCF-7	-9.47	*1.00*	-7.26	-2.26	3.17	*1.00*	5.71	5.52				
relative to MDA-MB-231	-8.08	3.65	*1.00*	2.83	-4.44	-8.25	*1.00*	*-0.33*				
relative to MDA-MB-468	-9.32	*1.29*	-6.46	*1.00*	-4.25	-8.19	*1.03*	*1.00*				

	**Uridine/UDP-G**											

relative to BT474	*1.00*	-6.83	-4.15	-4.52	*1.00*	-9.88	-8.88	-6.21				
relative to MCF-7	3.15	*1.00*	*1.84*	*1.73*	86.50	*1.00*	9.70	32.80				
relative to MDA-MB-231	*1.71*	-4.58	*1.00*	*-0.63*	8.92	-8.97	*1.00*	3.38				
relative to MDA-MB-468	*1.82*	-4.21	*1.07*	*1.00*	2.64	-9.70	-7.04	*1.00*				

	**Valine**											

relative to BT474									*1.00*	-4.12	*-0.75*	-2.48
relative to MCF-7									*1.70*	*1.00*	*1.57*	*1.28*
relative to MDA-MB-231									*1.08*	-3.65	*1.00*	*-1.88*
relative to MDA-MB-468									*1.33*	-2.18	*1.23*	*1.00*

Fold-change differences highlighted are magnitude ≥ ±2 for ^13^C intracellular (Table 1) and ^12^C production and consumption concentration (Table 2) comparisons relative to each cell line and between them. Concentration values of zero in Tables 1 and 2 were set to 1.00 for fold-change calculations. All fold-change differences less than ≥ 2-fold are in italics.

## Supplementary Materials

Supplementary Materials Supplementary file contains Supplemental Figure 1, demonstrating specified NMR spectral regions that are different across Luminal A and TNBC cell lines for (A) ^13^C-glucose or (B) ^13^C-glutamine metabolism.
