# Supplementary material for: Stable Isotope-Resolved Metabolomic Differences between Hormone-Responsive and Triple-Negative Breast Cancer Cell Lines
Source: Int J Breast Cancer. 2018 Sep 30;2018:2063540. doi: 10.1155/2018/2063540 (PMC6186330; doi:10.1155/2018/2063540)

**Supplementary Material**

**Supplemental Figure 1.** Differential cellular utilization of glucose and glutamine in Luminal A and TNBC cell lines. Full spectra and expanded regions show comparison of (**A**) ^13^C-glucose or (**B**) ^13^C-glutamine metabolism. Spectral chemical shifts are in ppm. **A.**

**B.**


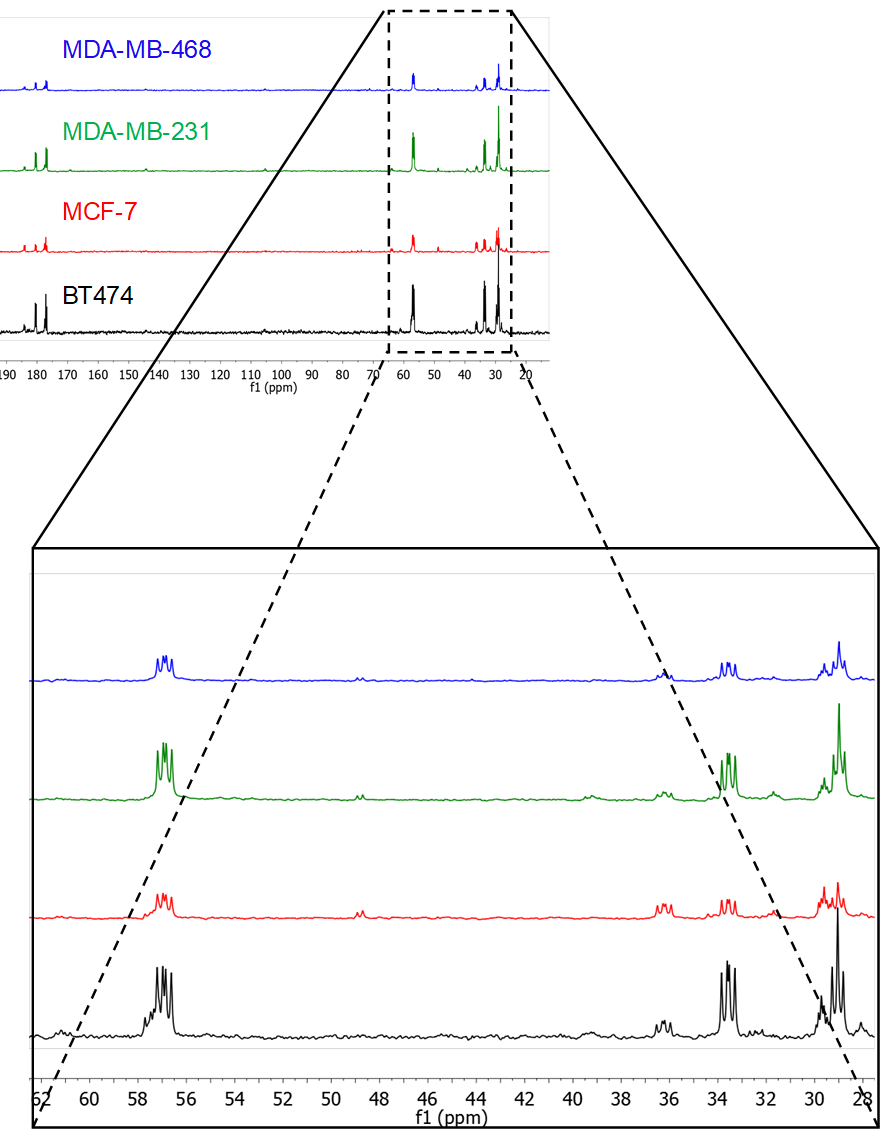

Supplement: Supplementary Materials — Supplementary file contains Supplemental Figure 1, demonstrating specified NMR spectral regions that are different across Luminal A and TNBC cell lines for (A) 13C-glucose or (B) 13C-glutamine metabolism. [file 2063540.f1.docx]
